# Cardio-respiratory signal extraction from video camera data for continuous non-contact vital sign monitoring using deep learning

**DOI:** 10.1088/1361-6579/ab525c

**Published:** 2019-12-02

**Authors:** Sitthichok Chaichulee, Mauricio Villarroel, João Jorge, Carlos Arteta, Kenny McCormick, Andrew Zisserman, Lionel Tarassenko

**Affiliations:** 1 Institute of Biomedical Engineering, Department of Engineering Science, University of Oxford, United Kingdom; 2 Visual Geometry Group, Department of Engineering Science, University of Oxford, United Kingdom; 3 Neonatal Unit, John Radcliffe Hospital, Oxford, United Kingdom; 4 Author to whom any correspndence should be addressed.; sitthichok.chaichulee@eng.ox.ac.uk

**Keywords:** deep learning, non-contact vital sign monitoring, neonatal intensive care unit, remote photoplethysmography, physiological monitoring, vital signs

## Abstract

Non-contact vital sign monitoring enables the estimation of vital signs, such as heart rate, respiratory rate and oxygen saturation (SpO_2_), by measuring subtle color changes on the skin surface using a video camera. For patients in a hospital ward, the main challenges in the development of continuous and robust non-contact monitoring techniques are the identification of time periods and the segmentation of skin regions of interest (ROIs) from which vital signs can be estimated. We propose a deep learning framework to tackle these challenges. *Approach*: This paper presents two convolutional neural network (CNN) models. The first network was designed for detecting the presence of a patient and segmenting the patient’s skin area. The second network combined the output from the first network with optical flow for identifying time periods of clinical intervention so that these periods can be excluded from the estimation of vital signs. Both networks were trained using video recordings from a clinical study involving 15 pre-term infants conducted in the high dependency area of the neonatal intensive care unit (NICU) of the John Radcliffe Hospital in Oxford, UK. *Main results*: Our proposed methods achieved an accuracy of 98.8% for patient detection, a mean intersection-over-union (IOU) score of 88.6% for skin segmentation and an accuracy of 94.5% for clinical intervention detection using two-fold cross validation. Our deep learning models produced accurate results and were robust to different skin tones, changes in light conditions, pose variations and different clinical interventions by medical staff and family visitors. *Significance*: Our approach allows cardio-respiratory signals to be continuously derived from the patient’s skin during which the patient is present and no clinical intervention is undertaken.

## 1. Introduction

Non-contact vital sign monitoring using a video camera enables the measurement of vital signs to be performed by measuring subtle color changes on the surface of the skin from a distance, without any sensors attached to the patient. Continuous non-contact monitoring of vital signs in a real-world hospital environment poses several challenges. The detection of time periods and skin regions of interest (ROIs) from which vital signs can be estimated is the particular focus of this paper. These tasks are important as they provide the essential information which enables the automatic extraction of vital signs from a video camera (Tarassenko *et al* 2014).

Studies in camera-based non-contact vital sign monitoring over the past decade have been carried out in controlled environments in which subjects remain relatively still throughout the video recording (Tarassenko *et al* 2014, Wieringa *et al* 2005, Verkruysse *et al* 2008, Wu *et al* 2012, Aarts *et al* 2013, Poh *et al* 2010, 2011, Scalise *et al* 2012). Several studies measured vital signs from ROIs which were manually selected and fixed across the video sequence (Wieringa *et al* 2005, Verkruysse *et al* 2008, Wu *et al* 2012, Aarts *et al* 2013). Many studies employed automated ROI selection methods in which face detection was used to define an ROI in the first frame and the ROI was then automatically tracked in consecutive frames using a feature tracker or an image segmentation algorithm (Tarassenko *et al* 2014, Poh *et al* 2010, 2011, Kumar *et al* 2015).

In the Neonatal intensive care unit (NICU), pre-term infants (infants born before 37 weeks of gestation) are active and clinical staff regularly interact with them. They are very impulsive and often move their arms and legs. Routine medical interventions are performed several times a day. Clinical interventions can cause severe motion artifacts which may prevent vital sign estimation from video camera data. Table 1 summarizes the clinical activities carried out by the nurses in the NICU. Typical clinical interventions are: checking the correct functionality of medical equipment, changing a diaper, taking temperature readings, administering medications and withdrawing blood from the heel for carrying out blood tests. Clinical staff or parents can remove infants from the incubator for kangaroo care (skin-to-skin cuddling). Light conditions not only change throughout the day, but also throughout the seasons of the year, with long daytime the summer and short daytime during the winter. Artificial light sources, such as overhead and room lights, may cause specular reflections from the skin surface of the infant and change the colors that are recorded by the video camera. Shadows are cast on the infant when clinical staff walk between these light sources and the incubator. These scenarios present challenges to the development of algorithms for the detection of appropriate time periods and ROIs in which vital signs could be estimated. To date, conventional processing methods have not been suitable for these tasks. New methods are required to be less dependent on skin tones, body postures, patient position and lighting conditions.

**Table 1.** Daily nursing activities for pre-term infants in the NICU (data provided by research nurses at the John Radcliffe Hospital).

Time	Event
At nurse shift handover (every 8–12 h)	– Lift incubator cover to examine the infant
	– Examine nasogastric tube (NGT) placement
	– Examine central venous line (CVL)

As required	– Check emergency equipment
	– Check ventilation equipment
	– Check fluid infusion pumps
	– Replace electrocardiogram (ECG) leads
	– Replace nasal probes

After bradycardia, O_2_ desaturation or apnea	– Provide tactile stimulation
	– Change infant position

Every hour	– Remove fluid from the airways
	– Give nasogastric tube (NGT) feed
	– Record vital sign parameters

Every 6 h	– Take temperature and blood pressure
	– Change skin probe sites
	– Change infant position

Every 12 h	– Take infant out of incubator for cuddles

Every 6–8 h, if under phototherapy	– Heel prick for a blood test

Every 2–6 h, if hypoglycemic	

Every 6–12 h	– Give oral medication

Every 4–12 h	– Give intravenous (IV) line medication

Convolutional neural networks (CNNs) have recently led to major advances in computer vision thanks to their ability to learn rich feature representations and combine feature generation and classifier training together (Krizhevsky *et al* 2012, Chatfield *et al* 2014, Simonyan and Zisserman 2015, Goodfellow *et al* 2016). CNNs are a computational model formed by multiple processing layers that can learn representations of data (Goodfellow *et al* 2016). Example applications in computer vision include image classification, image segmentation and action recognition.

The success of CNNs began with AlexNet (Krizhevsky *et al* 2012) with a network of five convolutional layers and two fully-connected layers designed for large-scale image classification. Simonyan and Zisserman (2015) demonstrated that network depth was an important factor for achieving high levels of performance. Their well-known network, VGG16, has 13 small convolutional layers and three fully-connected layers in a homogeneous arrangement. This network gained popularity through its good generalization on different datasets and different domains. Later, He *et al* (2016) increased the depth of the network up to 152 layers for image classification. Their networks, ResNets, used short connections to link non-successive intermediate layers together in order to help training very deep networks. For image segmentation, Long *et al* (2015) proposed fully convolutional network (FCN) models that extended the VGG16 network by up-sampling the output from intermediate layers or feature maps across the network to produce meaningful segmentation results. For action recognition, Simonyan and Zisserman (2014) introduced a two-stream CNN architecture, which consists of spatial and temporal processing streams designed for processing an individual video frame and time-varying optical flow vectors together for classifying the content of video recordings.

This work aims to answer three important questions arising prior to the estimation of vital signs in a hospital setting: (1) is a patient in the field of view of the camera? and (2) is a patient undergoing a clinical intervention? and (3) which pixels belong to skin? Vital signs could be estimated from ROIs on the patient’s skin only when the patient is present and no clinical intervention is being undertaken.

**Figure 1.**
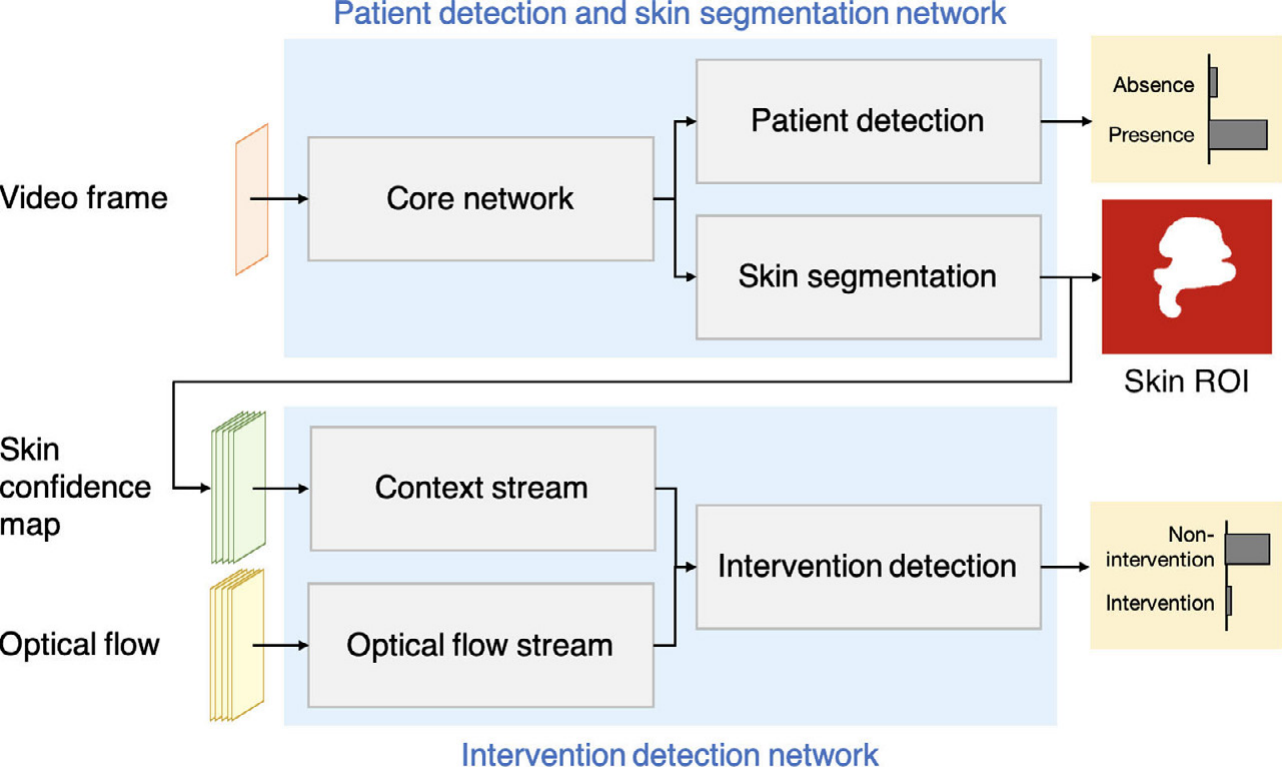
The proposed framework consists of two deep learning networks: the patient detection and skin segmentation network; and the intervention detection network. These networks operate in sequence to identify appropriate time periods and ROIs from which vital signs can be estimated.

We propose a deep learning framework to answer these questions (see figure 1). The approach consists of two CNN models working in sequence. The first network was designed for detecting the presence of a patient and segmenting the patient’s skin areas. A preliminary version of this network has been reported in Chaichulee *et al* (2017). The second network used the skin segmentation output from the first network with optical flow for identifying time periods of clinical intervention in the video data. We also show that photoplethysmographic imaging (PPGi) and respiratory signals can be derived using our deep learning framework. These signals can be used for the estimation of heart rate and respiratory rate.

This paper starts with the description of our clinical study in section 2. The deep learning model for patient detection and skin segmentation is described in section 3. The deep learning model for intervention detection is described in section 4. The evaluation protocol is described in section 5. The performance of both networks is reported in section 6, followed by a discussion of their performance in section 7. A conclusion is presented in section 8.

## 2. Clinical study

This work was carried out on data from a clinical study which was designed to investigate the use of video cameras for monitoring the vital signs of 30 pre-term infants. The clinical study was conducted in the high-dependency area of the NICU at the John Radcliffe Hospital, Oxford, UK. The pre-term infants were double-monitored with a digital video camera and the standard patient monitoring devices, without interrupting normal patient care. Each pre-term infant was recorded under regular ambient light during daytime for up to four consecutive days. The study was approved by the South Central—Oxford A Medical Research Ethics Committee (MREC) under the reference number 13/SC/0597.

**Figure 2.**
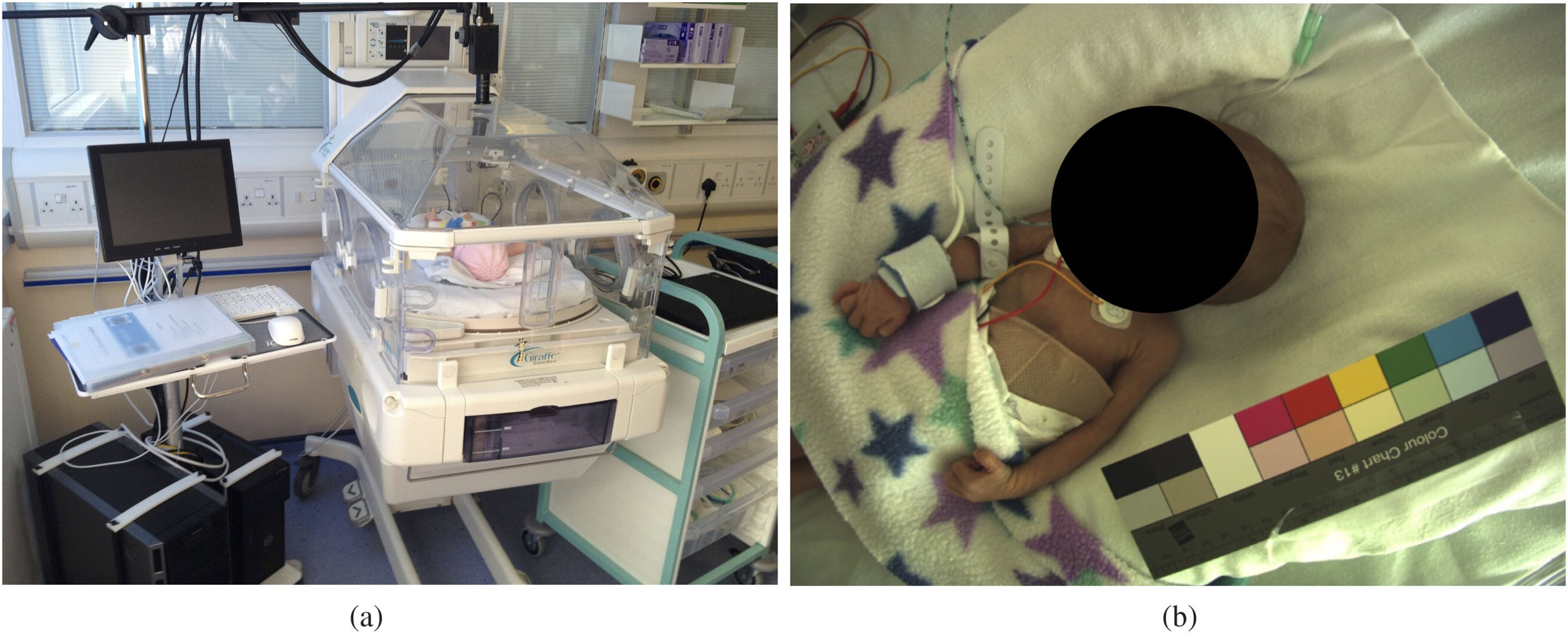
Equipment set-up for video recording: (a) camera, recording workstation and incubator; and (b) sample video frame.

The video recordings were acquired using a 3-CCD JAI AT-200CL digital video camera (JAI A/S, Denmark). The video camera has three Sony ICX274AL charge-coupled device (CCD) 1/1.8’ image sensors (Sony, Japan) to measure the light intensity of three color channels (red, green and blue) separately. The camera was equipped with a VS Technology SV-0614H lens (VS Technology, Japan). The video camera system acquired 24-bit raw uncompressed color images (8-bit per color) at a resolution of $1620\times1236$ pixels and a sampling rate of 20 frames per second. The functionalities related to the automatic adjustments of the video camera (such as auto-focus, auto-exposure and auto-white balance) were not applied. No constraints were imposed on the infant’s posture, position and orientation. Figure 2 shows the data acquisition set-up and sample images from the clinical study.

The recruitment of participants had no restrictions on infant weight, ethnic groups or skin tones. The dataset comprises a total of 90 recording sessions from 30 pre-term infants. A more detailed description of the study can be found in Villarroel *et al* (2014).

The clinical study protocol allowed the algorithms to be developed and validated on half the participants and then to be tested on the other half. Therefore, the participants were divided into two groups of 15 infants. This paper used only video data from the first group. Table 2 provides a summary of the demographics of the participants.

**Table 2.** Summary of population demographics.

Total participants	15
Total recording sessions	43
Gestational age^a,b^ (weeks)	$28.9 (\pm3.2)$
Weight^a,b^ (grams)	$1172.2 (\pm284.3)$
Gender (number of participants)	
Male	8
Female	7
Ethnicity (number of participants)	
White British	10
Asian or Asian British—Pakistani	1
Black British or black African	1
Mixed—White and Asian	1
Mixed—White and black Caribbean	1
Mixed—Any other mixed	1

a Value shown in mean ($\pm$SD).

b On the first day of recording.

## 3. Patient detection and skin segmentation network

**Figure 3.**
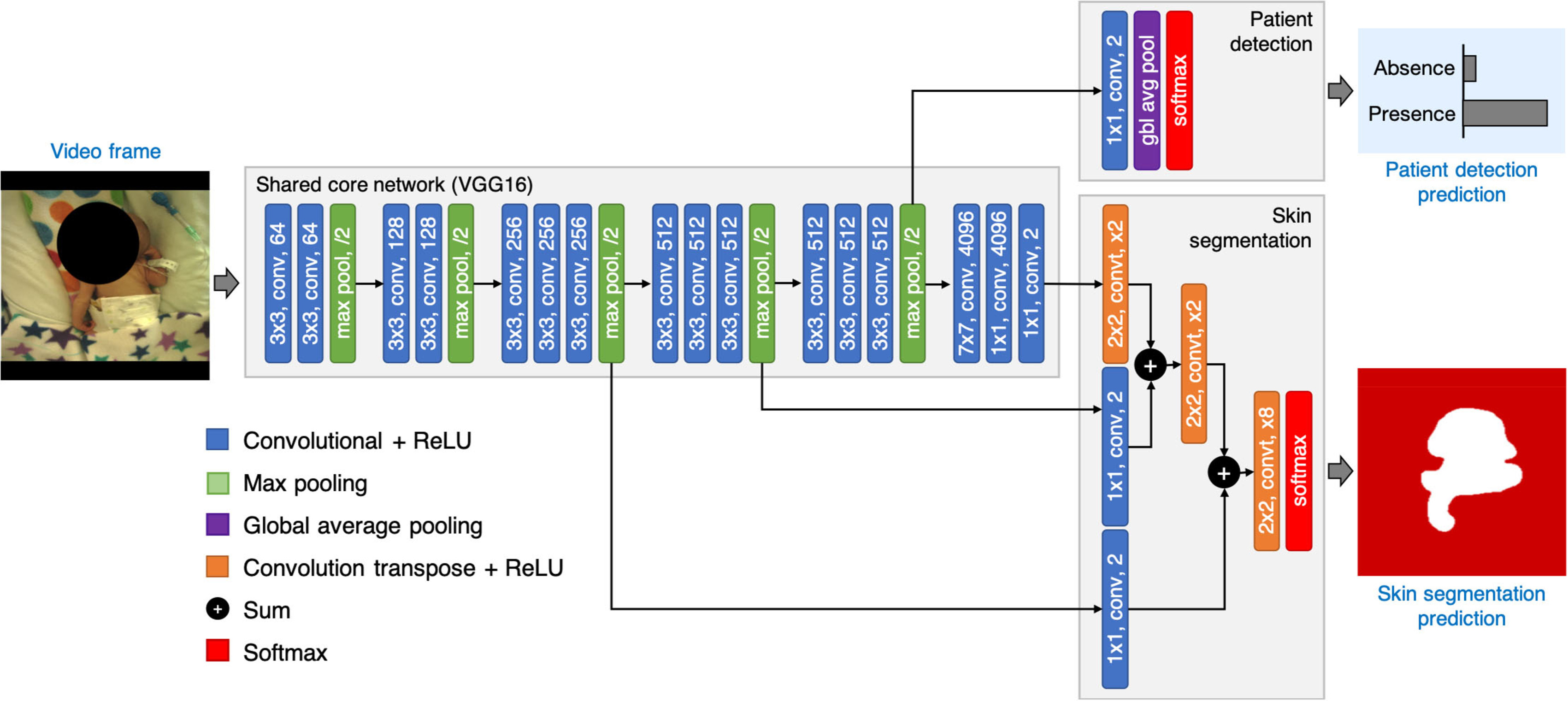
The proposed patient detection and skin segmentation network has two output streams. The patient detection stream performs global average pooling over feature maps to predict the presence of the patient in the scene. The skin segmentation stream performs hierarchical upsampling of feature maps across the shared core network to produce a skin label. The network was designed to evaluate the skin segmentation stream only if the infant was present in the scene.

This section describes the patient detection and skin segmentation network which is the first network in our proposed framework (see figure 1). The network was developed for detecting the presence of the infant in the scene and segmenting the infant’s skin regions. Our network receives a single video frame and produces two outputs: a patient detection score and a skin label (see figure 3).

### 3.1. Network architecture

The patient detection and skin segmentation problems were formulated as a joint task of image classification and segmentation built into a single multi-task model (see figure 3). Our network has a shared core network with two output streams: the patient detection stream which was implemented using global average pooling; and the skin segmentation stream which was implemented using hierarchical upsampling of feature maps across the shared core network. The patient detection stream was set to perform first, followed by skin segmentation only if a patient was found in the video frame. The skin segmentation stream was not processed further when the patient was absent from the field of view.

#### 3.1.1. Shared core network

The shared core network was used to extract image features that are common between the two tasks. The core network was implemented based on the VGG-16 network, proposed by Simonyan and Zisserman (2015). The VGG-16 network has been recognized as a generic feature extractor and has demonstrated good generalization in transfer learning towards other datasets.

The VGG-16 architecture is made up of a stack of $3\times3$ convolution layers for extracting image features with rectified linear unit (ReLU) layers for highlighting distinct features and periodically followed by $2\times2$ max-pooling layers for downsampling feature maps by a factor of two. The structure repeats until the output has a small spatial size and a decision is made upon that output by a set of three fully-connected layers for high-level reasoning and a softmax layer for calculating class scores.

Our extension to the VGG-16 network follows that of the FCN model introduced by Long *et al* (2015). The original VGG-16 network produces class scoring estimates. Several modifications were needed to enable pixel-level segmentation from the output of the shared core network. All fully-connected layers in the VGG-16 network were converted into convolution layers by having them perform convolution operations by sliding their filters across the input data. These layers were then able to produce a spatial output map with spatial coordinates preserved. The last convolution layer was modified to produce 2-class scoring outputs for the non-skin and skin classes.

#### 3.1.2. Patient detection

The patient detection stream was implemented using global average pooling for image classification similar to Lin *et al* (2014) and Szegedy *et al* (2015). A $1 \times 1$ convolutional layer with two outputs was added on top of the last max-pooling layer in the shared core network to perform linear combination and reduce the dimensionality of feature maps. A global average layer was then added on top of the $1 \times 1$ convolutional layer to average out the spatial information. The resulting output vector was then fed to a softmax layer for calculating class confidence estimates for the absence and presence of the infant.

#### 3.1.3. Skin segmentation

The skin segmentation stream was implemented based on the FCN model (Long *et al* 2015), which performs a series of learnable spatial upsampling operations to project feature maps across the network onto a larger-dimensional space in order to produce pixel-level segmentation of the skin (see figure 3).

The feature maps of the last convolutional layer in the shared core network contained coarse predictions for non-skin and skin classes at a subsampling factor of 32. A prediction at higher resolution could be obtained by combining the coarse predictions of this layer with the finer predictions of shallower layers. Each $1 \times 1$ convolutional layer with two outputs was added on top of the fourth and third max-pooling layers to produce two additional predictions of non-skin and skin classes at finer resolutions (at subsampling factors of 16 and 8 respectively). The prediction from the last convolutional layer was then spatially upsampled using a convolution transpose layer with an upsampling factor of 2 and combined with the prediction of the fourth max-pooling layer. Similarly, the combined prediction from the last convolutional layer and the fourth max-pooling layer was spatially upsampled by a factor of 2 and then combined with the prediction of the third max-pooling layer, resulting in a prediction at a factor of 8 of the original resolution. Lastly, a convolution transpose layer with an upsampling factor of 8 was added in order to obtain a final prediction at the same spatial size as the input image. The network was completed with a softmax layer that produced per-pixel class scoring estimates (or skin confidence map). A threshold was applied to confidence estimates to produce a skin label.

### 3.2. Training data

#### 3.2.1. Skin annotation

Skin annotation is a process of labeling skin areas in an image in order to create a dataset for training a CNN model. Although changes in daytime lighting conditions can affect the color of the skin perceived by the video camera, sudden changes rarely occur. Therefore, it is not necessary to annotate every frame in a video recording since multiple consecutive frames mostly contain similar information. In this work, three human annotators were asked to independently annotate skin regions in a representative number of frames extracted from the recorded videos.

**Figure 4.**
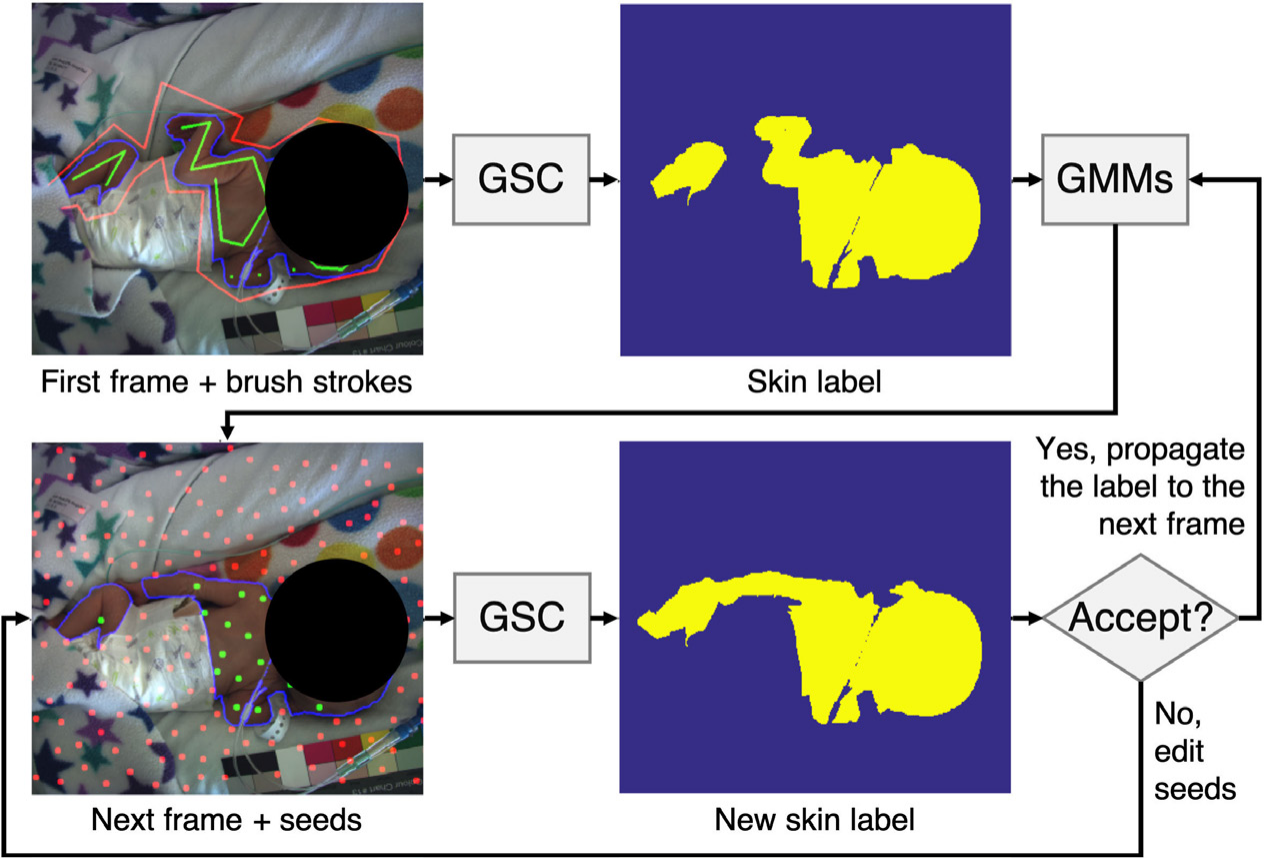
Flowchart of semi-automatic skin annotation. Each annotator was asked to label skin areas in the first image of each session. The label was then propagated to the next frame using GMMs. The annotator can interact with seeds (green and red circles corresponding to skin or non-skin areas respectively) to modify the skin label for the new image frame.

Due to the large size of the video recording, we developed a customized skin annotation tool to reduce the human effort required for manually labeling skin regions. The algorithm for the semi-automatic skin annotation process was developed using the graph-cut segmentation with geodesic star convexity (GSC) introduced by Gulshan *et al* (2010). The block diagram outlining this semi-automatic approach is shown in figure 4. For each recording session, the annotators were asked to label skin regions in the first image. The skin label was then propagated to the next image where the annotators could adjust the skin label.

The GSC algorithm (Gulshan *et al* 2010) utilizes geodesic star shape priors that exploit both color and image gradient data to represent shapes. The algorithm requires a human user to first lay out hard constraints for the segmentation procedure by creating brush strokes on non-skin and skin areas. These brush strokes are used for creating color models and for exploiting image structure through geodesic stars. The algorithm then generates a skin label containing a partition between skin and non-skin regions in the image based on energy minimization subject to geodesic star-convexity constraints.

The skin label was propagated to the subsequent image using Gaussian mixture models (GMMs), which learned the color properties of non-skin and skin regions from the previously annotated skin labels. For the next image, the GMMs produced the probability of each pixel belonging to skin. These probabilities were used to generate segmentation seeds, which are simulated brush strokes. The skin segmentation seeds were produced in areas with high skin probabilities, while the non-skin segmentation seeds were produced in areas with high non-skin probabilities. The location of the segmentation seeds were determined by Mitchell’s best candidate algorithm (Mitchell 1991). Skin labels were then computed using the GSC algorithm with these segmentation seeds. The skin labels could be altered by modifying the segmentation seeds if the annotators were not satisfied with the result. The segmentation seeds provide an effective way for the annotators to interact with automatically-generated skin labels.

Skin annotation was performed by three annotators to ensure that high-quality ground truth data were obtained. Taking into account the trade-off between annotation effort and robustness to lighting variations, one video frame was extracted every 6 min from each video recording. So, 2269 images were obtained covering a total recording time of 226 h. All annotators were asked to label the same set of images.

The annotators were asked to skip the annotation when: the image did not contain an infant, the scene was too dark to segment the skin, clinical staff or parents were present in the image, or the infant was undergoing phototherapy treatment (infants treated for jaundice by exposure to high-intensity blue light).

Even though the semi-automated approach was employed for skin annotation, all images were reviewed and all skin annotation labels were confirmed by all annotators. Once finished, the skin labels from all annotators were then combined in the following manner. Images were considered as *positive* if more than two human annotators provided skin labels, without skipping the images. This criterion has resulted in a total of 1718 out of 2269 images (76.0%) were considered as *positive*. For each image, pixels were considered as skin if two or more annotators agreed upon, otherwise pixels were considered as non-skin. The agreement of skin annotation was computed using the ratio of the intersection of skin labels provided by at least two annotators to the union of those provided by three annotators. The mean skin annotation agreement was 96.5%.

#### 3.2.2. Patient annotation

This dataset contains *positive* images in which the infant was present and pixel-level skin annotation was provided, and *negative* images, representing video frames in which the infant was absent. These labels were used to train the CNN model for the patient detection task.

During the studies, infants were often taken from the incubator for kangaroo care (skin-to-skin contact with their mother) or for other clinical interventions. There was a designated quiet period of about 6 h for every 24 h during which the lights were dimmed or the incubator was covered to allow the infant to rest. Negative images were annotated by the three annotators on the images that were collected from these periods, as reported in clinical records.

For the 15 infants, these periods accounted for 23.5 out of 226 h of video recordings. Images were taken every 20 seconds during these periods. This results in 4227 images for the annotators to decide. The same annotation strategy, the agreement of two out of three annotators, was utilized for the negative set. All the images were presented to the same set of annotators. Each annotator needed to identify each image as infant or non-infant. The images with at least two agreements considered as non-infant were regarded as negative. So, 2885 negative images were obtained. In order to create a balanced dataset, 2885 negative images were randomly downsampled to 1718 negative images.

### 3.3. Network training

#### 3.3.1. Data preparation

All training images and ground truth labels were resized to $512 \times 512$ pixels using bilinear interpolation with the aspect ratio maintained by allowing black spaces at the top and bottom of each image. Data augmentation, as explained below, was applied to improve generalization in the training set. Mean subtraction was then performed by calculating the mean value of each color channel across all images in the training set and subtracting the mean value from each image in order to center the data around the origin. This process was reported to improve the numerical condition of the network during training (LeCun *et al* 2012).

#### 3.3.2. Data augmentation

**Figure 5.**
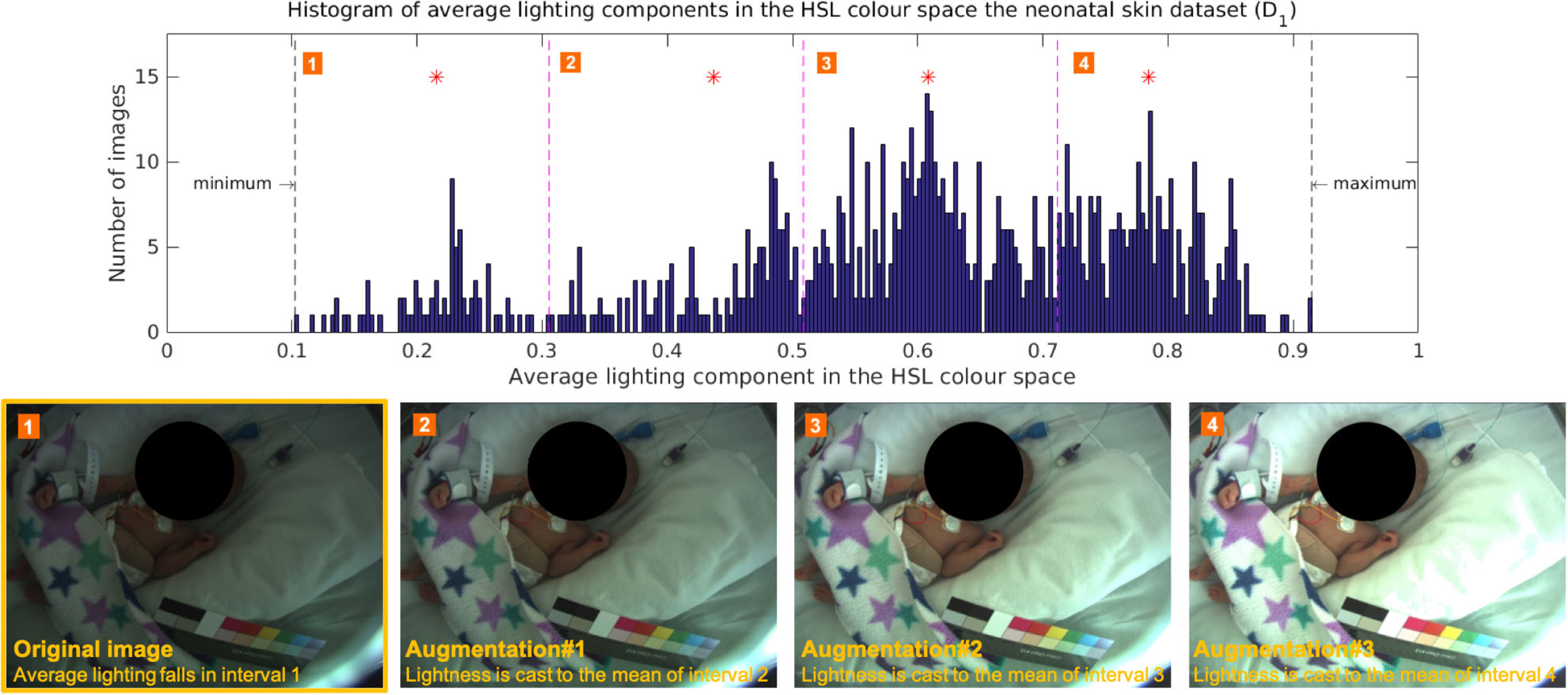
Lighting augmentation was applied to generate additional training images with different lighting conditions. The histogram of the average lighting components of all training images was divided into four uniform intervals. The mean of each interval was computed (marked with a red asterisk). Three additional images were generated by scaling the lighting component of the original image to the mean of the interval 2, 3, and 4 respectively.

Data augmentation is a method utilized to reduce overfitting and improve the generalization of the deep learning network. Multiple variations of each training image were generated. We employed three augmentation techniques to create more training data:

• *Lighting variations*: Lighting conditions fluctuated a lot during the daytime as a result of changes in both natural and artificial light sources. By varying the lighting characteristics in each image, the network could be made invariant to illumination changes. Three additional images were generated for each original image by scaling the lightness component in the HSL color space (see figure 5).
• *Rotation*: The position of the video camera and the orientation of the infant in the incubator can change at the discretion of clinical staff. To encourage the network to learn rotational invariance, for each original image, seven additional images were generated by rotating the original image at 45-degree increments throughout 360 degree rotation without resizing.
• *Mirroring*: To encourage the network to learn the symmetry of the human body, two additional images were generated by mirroring each original image with respect to the center of the image on the *x*-axis and *y* -axis.

In addition to the generation of more training data, random translation with a displacement factor between $-10\%$ and $10\%$ in both vertical and horizontal directions and random resizing with a scaling factor between 0.90 and 1.10 were also applied to the images during training.

#### 3.3.3. Loss functions

Each output stream was associated with an individual loss function. The patient detection branch was equipped with a multinomial logistic loss, as in Lin *et al* (2014) and He *et al* (2016), which measures the discrepancy between the softmax estimates produced by the patient detection stream and the associated ground truth labels. The skin segmentation branch was equipped with a multinomial logistic loss, similarly to Long *et al* (2015). The loss was summed across all spatial pixels and normalized according to the proportion of ground truth non-skin pixels and ground truth skin pixels. The unified multi-objective loss function was the weighted sum of these two individual losses.

#### 3.3.4. Model initialization

Batch normalization layers were first added between a convolutional layer and a ReLU layer in the shared core network. Batch normalization was employed to normalize the output of the convolutional layer to have a zero mean and unit variance by using the statistics computed from the entire batch. It can improve the generalization of the network and reduce training time by enabling the use of a high learning rate (Ioffe and Szegedy 2015).

The share core network was initialized with the original weights of the VGG16 network which hold the accumulated knowledge on edges, shapes, and patterns. All new convolutional layers were initialized with the Xavier algorithm (Glorot and Bengio 2010) with zero bias. The Xavier initialization created a reasonable range of weight values uniformly distributed across the layer. As suggested by Long *et al* (2015), convolution transpose layers were initialized using bilinear interpolation filters with no bias.

#### 3.3.5. Training procedures

The CNN network was implemented using the MatConvNet framework (Vedaldi and Lenc 2015). The training was performed using standard Stochastic Gradient Descent (SGD) optimization in two stages. The network was first trained for the skin segmentation task using only the positive images with annotated skin labels. All the weight layers of the shared core network and the skin segmentation stream were updated. The learning rates started at 10^−2^ and dropped by a factor of 10 every two epochs until convergence, with a batch size of 20 and a momentum of 0.90. The network was subsequently trained jointly for the patient detection and skin segmentation tasks using both the positive and negative images. All the weight layers of the network were updated. The individual loss function for each task was weighted equally. The learning rates started at 10^−4^ and dropped by a factor of 10 every two epochs until convergence, with a batch size of 20 and a momentum of 0.90. Once the training was completed, a computation sequence was arranged so that skin segmentation was performed only when the infant was present in the image.

## 4. Intervention detection network

**Figure 6.**
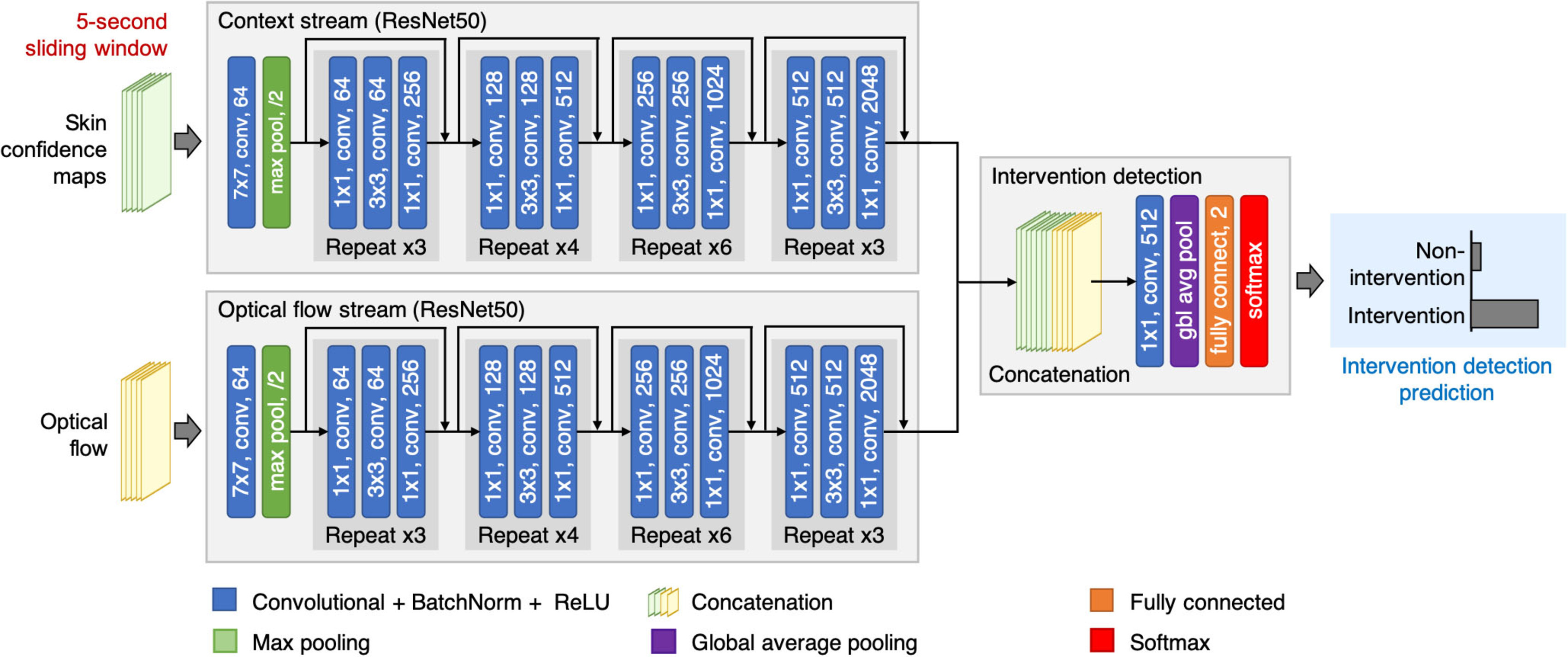
The proposed intervention detection network operates on a 5 s time window. The network consists of two input streams. The first input stream (context stream) processes a stack of skin confidence maps, produced by the patient detection and skin segmentation network. The second input stream (optical flow stream) handles a stack of dense optical flow. The outputs from both input streams are then combined to predict the occurrence of a clinical intervention in a given time window.

Our proposed framework consists of the patient detection and skin segmentation network and the intervention detection network working in sequence (see figure 1). This section describes the intervention detection network which is the second network in our proposed framework. The network takes multiple-frame skin confidence maps produced by the patient detection and skin segmentation network and multiple-frame optical flow vectors to identify the occurrence of clinical interventions in the video stream (see figure 6).

The skin confidence map contains a confidence value for each pixel belonging to the skin in the video frame. It is defined as the softmax output of the skin segmentation branch of the patient detection and skin segmentation network. Optical flow is a vector field describing the apparent motion of pixels between two images. It is comprised of the displacement vectors of points from the first image to the second image in the horizontal and vertical directions (Brox *et al* 2004).

### 4.1. Sliding window operation

**Figure 7.**
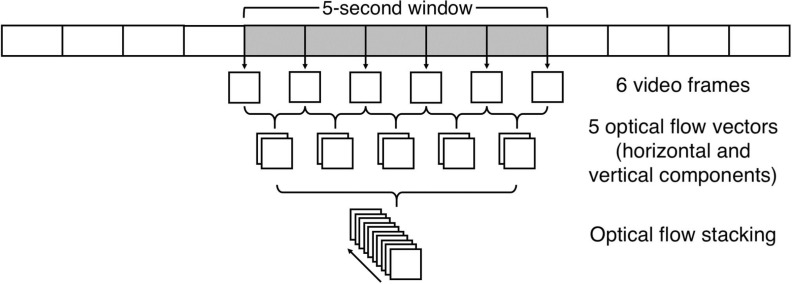
The processing of the input to the optical flow input stream. For each time window of 5 s, six video frames were taken, one image per second. A total of five optical flow vectors were computed from each pair of consecutive video frames. The horizontal and vertical components of each optical flow were then stacked together.

In order to identify intervention periods in long video recordings (6–8 h per video), a sliding-window approach was used to process the video sequence with a window length of 5 s and a step size of 1 s. From this sliding window, six video frames were extracted, one image every second (see figure 7).

For each extracted video frame, a skin confidence map was computed from the patient detection and skin segmentation network. The resulting six skin confidence maps were then stacked across an input channel. Subsequently, five optical flow fields were extracted; one for each pair of consecutive video frames in the sequence (see figure 7). The horizontal and vertical components of each optical flow vector are then stacked together across an input channel.

### 4.2. Network architecture

The intervention detection network has two input streams (see figure 6). Each stream was implemented using the pre-trained ResNet50 network with 50 weighted layers, as proposed in He *et al* (2016). Even though the number of weighted layers in the ResNet50 network is higher than the VGG16 network, each layer in the ResNet50 network is small and has fewer parameters.

For the first stream (the context stream), the ResNet50 architecture was modified to take six multiple-frame skin confidence maps as input, derived from the patient detection and skin detection network. The first convolutional layer was extended to six channels by stacking the spatial average of the original first convolutional filters across the channels. The average pooling layer and fully-connected layer (the last two layers of the ResNet50 network) were removed.

For the second stream (the optical flow stream), the ResNet50 architecture was modified similarly to the context stream. Instead, it took ten multiple-frame optical flow vectors as input, derived using the algorithm proposed by Brox *et al* (2004). The network was modified in a similar manner as the context stream.

A decision on intervention detection was made upon the output of both input streams. The feature maps from the last convolutional layer of both input streams were fused together through a series of concatenation and convolutional layers as in Feichtenhofer *et al* (2016a). Both output feature maps were first stacked together and then convolved with a $1\times1$ convolutional layer with 512 outputs. The convolutional layer performed a weighted combination of feature maps from both branches and reduced the dimension of the combined feature channels. This layer could learn information corresponding to a decision-making process from both networks (Feichtenhofer *et al* 2016a). The network ended with a global average pooling layer, a fully-connected layer with two outputs and a softmax layer. The last layer provided classification scores to distinguish between intervention and non-intervention events.

### 4.3. Training data

The training of the intervention detection network requires a dataset of annotated intervention periods. Such information was not provided in full by the clinical staff during the clinical study. To obtain training data, the start and end time points of three mutually exclusive events were annotated in the video dataset: intervention, non-intervention and infant absence.

The same three human annotators were employed. This approach eliminated the chance of missing annotations which might have occurred when a single annotator was fatigued and overlooked the occurrence of an event. All video sessions in the 15-infant dataset were annotated. Similar to the training data of the patient detection and skin segmentation network, the periods during which the infant was under phototherapy were excluded from the annotation. Therefore, the annotators were required to label the periods of intervention, non-intervention and baby absence for a total of 214.0 h of video.

A specialized annotation tool was developed. Annotators were asked to watch videos played at 30 times the original speed. This was to ensure that the videos were seen by the annotators in a reasonable amount of time. They could navigate forward and backward in time. However, forward navigation was not allowed unless the video section had been previously watched. The annotators were asked to mark the start and end frame numbers in the video for sections during which medical staff or parent(s) were present in the video frame (intervention), the baby was present in the video frame without medical staff or parents (non-intervention) and the baby was not present in the frame (infant absence).

The intervention labels provided by the three annotators were combined based on the consensus among the annotators. Subsequently, the annotations, which were performed at the frame level, were converted into one-second labels using the consensus among the labeled video frames for each second.

The Fleiss’ kappa inter-rater reliability of agreement between the three annotators was 96.1. Of the 214.0 h of annotated videos, 178.9 h were marked as non-intervention, 16.7 h were marked as intervention and 18.4 h were marked as baby absence periods.

### 4.4. Network training

#### 4.4.1. Data preparation

Skin confidence maps and dense optical flows were pre-computed for each second in the videos using the optical flow algorithm of Brox *et al* (2004). Optical flows were stored in compressed JPEG image files as in Simonyan and Zisserman (2014) in order to reduce storage space and allow the dataset to be stored in memory. Since the optical flows were stored in an 8-bit integer format, the values can only vary from 0 to 255. For each optical flow component, displacement values bigger than 40 pixels were clipped to 40 pixels as the displacement of more than 40 pixels over one second rarely happened. These values were then scaled to the range between 0 and 255, similarly to Feichtenhofer *et al* (2016b).

#### 4.4.2. Data augmentation

Data augmentation was performed to add variations to the training data. The following image transformation techniques were applied to all images in both the skin confidence map and optical flow stacks during training.

• *Random resizing*: To let the network learn about different sizes of the subject, images were randomly resized between scales of 1.00 and 1.25.
• *Random translation*: To encourage the network to learn that the patient can be positioned anywhere in the field of view, images were randomly translated between  −10% and 10% in both vertical and horizontal directions.
• *Random rotation*: As the patient’s orientation can vary spontaneously, images were randomly rotated between $-45^{\circ}$ and $45^{\circ}$.
• *Random mirroring*: Since humans have body symmetry, training images were mirrored vertically and horizontally by random.

#### 4.4.3. Training procedures

The CNN models were implemented on the MatConvNet framework (Vedaldi and Lenc 2015). All new layers were initialized with the Xavier algorithm with zero bias (Glorot and Bengio 2010). The network’s final layer was equipped with a multinomial logistic loss function.

The sliding window approach resulted in a large amount of short video segments. The training was performed only on the periods that were labeled as intervention and non-intervention. Each sliding window was regarded as intervention if more than half of the time was labeled as intervention, otherwise it was regarded as non-intervention. Each sliding window segment was treated as an independent and separate sample.

The training was performed using standard stochastic gradient descent in two stages in order to reduce training time and avoid overfitting. Mean subtraction was first applied to zero-center the network input as in Simonyan and Zisserman (2014). For each training iteration, video segments were sampled uniformly across intervention and non-intervention classes to create a balanced training set.

In the first stage, both context and optical-flow streams were trained individually, while keeping the average pooling and fully-connected layers, with a momentum of 0.90 and a batch size of 24 samples. During training, all the weight layers of each individual network were updated. The learning rates were scheduled to start at 10^−3^ and reduced by a factor of ten for every 12 000 iterations until convergence.

In the second stage, the intervention detection network was formed. The training was performed only on the new weight layers added after fusion with a momentum of 0.90, a batch size of 12 and a learning rate of 10^−5^. The learning rate was decreased by a factor of 10 for every 6000 iterations until convergence.

## 5. Evaluation protocol

Predictive performance was obtained using cross-validation on two independent groups. The dataset of 15 infants was first divided into two groups, *D*_1_ and *D*_2_. The *D*_1_ group had eight subjects and the *D*_2_ group had the other seven subjects. The assignment to each set was based on a balance of video recording information (date, time and duration) and participant demographics (ethnicity, gestational age and weight). Table 3 gives a summary of participant demographics for the *D*_1_ and *D*_2_ set. The two-fold cross-validation process requires training and validating the model two times with different data. The first model was trained on *D*_1_ and validated on *D*_2_. Then, the other model was trained on *D*_2_ and validated on *D*_1_. Hence, that the images that were used to validate the network were taken from different infants from the images that were used to train the network. The validation results from both models were combined to calculate overall predictive performance. The results of our proposed algorithms were then compared with those obtained using other baseline methods.

**Table 3.** Summary of patient demographics in the *D*_1_ and *D*_2_ sets.

Set	Subjects	Sessions	Hours	Gender^a^		Ethnicity^b^					
				Male	Female	White	Black	Asian	WB	WA	Other
				
*D*_1_	8	22	118.7	5	3	5	1	1	—	—	1
*D*_2_	7	21	107.7	3	4	5	—	—	1	1	—

Total	15	43	226.4	8	7	10	1	1	1	1	1

a WB  =  Mixed White and Black,

b WA  =  Mixed White and Asian.

### 5.1. Patient detection and skin segmentation

Baseline experiments for patient detection and skin segmentation tasks were conducted using color-based skin color filters, implemented using three classifiers: naive Bayes (Jones and Rehg 2002), random forests (Breiman 2001) and GMMs (Bishop 2006). The skin filters classify each pixel in the image as a skin pixel based on skin colors and provide a skin confidence map, which can be later thresholded to a binary skin label. The skin filters were trained on images that were converted to the HSL (hue-saturation-lightness) color space (Kakumanu *et al* 2007) with white balance correction applied (Bianco and Schettini 2010). Patient detection was performed using the ratio of skin to non-skin pixels and the average confidence of predicted skin pixels to make a decision, as in the method described in Jones and Rehg (2002).

### 5.2. Intervention detection

A baseline experiment for clinical intervention detection was implemented using the two-stream deep learning architecture for action recognition proposed by Simonyan and Zisserman (2014) in which the outputs of the two network streams were combined using the support vector machine (SVM) technique.

## 6. Results

### 6.1. Patient detection

For patient detection, the classifiers performance is described using the receiver operating characteristics (ROC) curve, accuracy, recall, precision and specificity. The area under the ROC curve (AUC) provides a measure to describe overall performance.

Table 4 shows the results for the patient detection task. Similar performances are reported for all the baseline methods and the proposed CNN model. The Naive Bayes model obtained the highest precision and specificity of $97.8\%$ and $97.8\%$ respectively. The GMMs model achieved the highest AUC, precision and specificity of $98.8\%$, $97.8\%$ and $97.8\%$ respectively. The proposed CNN model has the highest accuracy and recall of $98.8\%$ and $100.0\%$ respectively. The proposed model achieved an AUC of $98.2\%$.

**Table 4.** Patient detection results.

Model	AUC	Accuracy	Precision	Recall	Specificity
Baseline methods					
Naive Bayes	98.1	98.6	**97.8**	99.4	**97.8**
Random forests	97.2	97.7	97.5	97.9	97.4
GMMs	**98.8**	97.1	**97.8**	96.4	**97.8**
Proposed CNN	98.2	**98.8**	97.6	**100.0**	96.8

*Note*. All performance values are expressed as a percentage.

### 6.2. Skin segmentation

**Figure 8.**
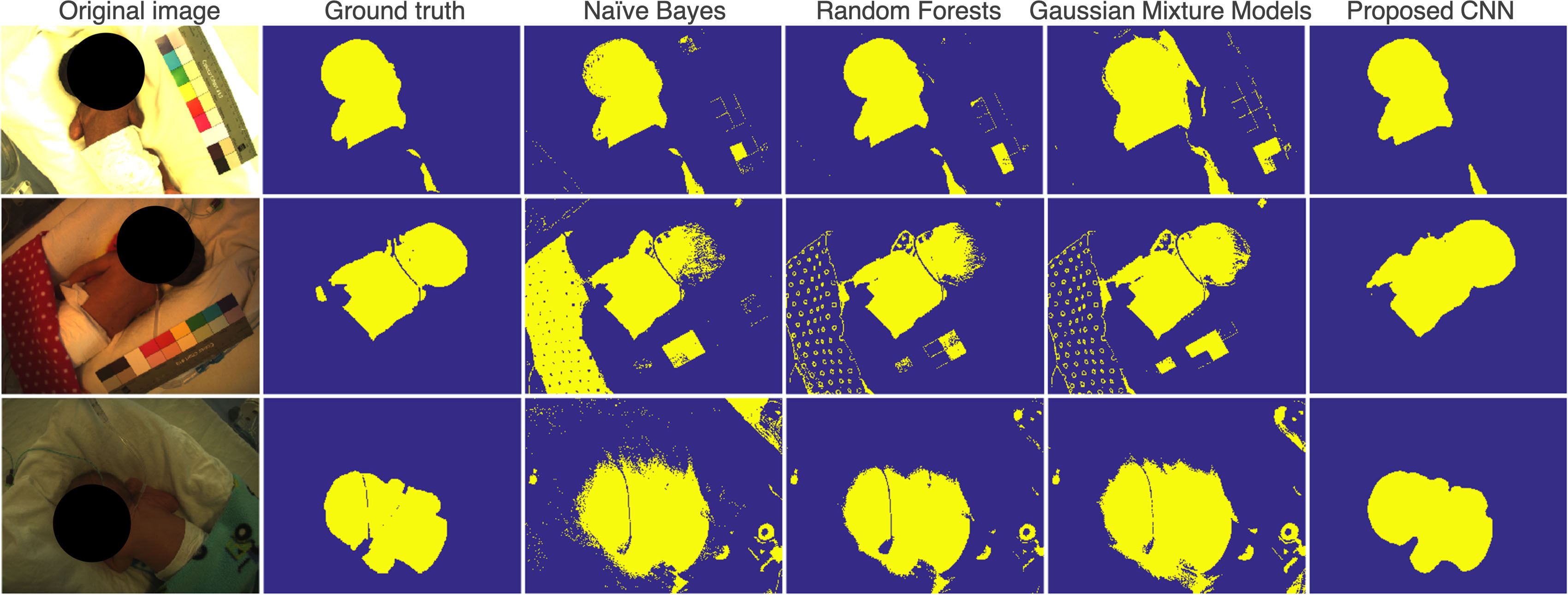
Example images for skin segmentation results.

For skin segmentation, the method of evaluation was intersection over union (IOU) which quantifies the proportional overlap between a target and a segmentation output. Table 5 shows the results for skin segmentation tasks. The proposed CNN model outperformed all the baseline methods for all metrics. The proposed CNN model yielded a mean IOU of $88.6\%$ and a mean pixel accuracy of $98.1\%$. Figure 8 shows example skin segmentation results on typical video frames from the clinical study.

**Table 5.** Skin segmentation results.

	Pixel accuracy			Intersection over union		
Model	Mean (SD)	Min	Max	Mean (SD)	Min	Max
Baseline methods						
Naive Bayes	89.5 (8.3)	32.7	98.9	61.3 (17.4)	4.3	92.9
Random forests	95.0 (4.6)	57.7	99.3	75.9 (16.1)	6.8	95.4
GMMs	93.4 (5.2)	47.5	99.1	71.2 (14.2)	16.8	94.7
Proposed CNN	**98.1 (1.9)**	**75.6**	**99.6**	**88.6 (7.5)**	**39.0**	**97.0**

*Note*. All performance values are expressed as a percentage.

Performance are evaluated on images with the presence of a subject.

### 6.3. Intervention detection

Table 6 shows the results for intervention detection. When the skin confidence stream and the optical flow stream were trained individually (after the first training stage), their performances were slightly lower than those of the baseline method. When both streams were combined, the network achieved the highest AUC of $98.2\%$ with the highest accuracy of $94.5\%$. The proposed CNN model yielded better results than the baseline method (Simonyan and Zisserman 2014) for all metrics.

**Table 6.** Intervention detection results.

Model	AUC	Accuracy	Precision	Recall	Specificity
Baseline method	98.1	92.4	90.8	94.4	90.5
Proposed CNN					
Skin confidence stream	95.9	89.7	89.6	89.9	89.5
Optical flow stream	97.4	92.2	91.7	92.8	91.6
Fusion network	**98.2**	**94.5**	**94.4**	**94.7**	**94.4**

*Note*. All performance values are expressed as a percentage.

## 7. Discussion

Our proposed CNN model correctly segmented skin regions under normal ambient light (see figure 8). The model had some difficulties with low-light images. However, it performed better than the color-based skin filters. In most of the images for which an infant was absent, our proposed CNN model did not produce a skin label. The baseline methods produced a skin label with salt noise and unwanted pixel groups. This could be improved with morphological operators, such as dilation and erosion. The CNN model processed the whole image at once and it did not need a further post-processing step. However, the CNN model could not identify small skin regions as the architecture periodically downsamples feature maps in the network.

Different sliding window sizes were examined when designing the intervention detection network. A sufficiently large sliding window length is required to capture motion information over video frames. However, a very large window length can cause deterioration in performance as it may lead to overestimation of the start and end times of an intervention interval. Table 7 shows the performance of the optical flow stream trained individually with different sliding window configurations. A window length of *T*  =  5 s and a step size of $\tau = 1$ s achieved the highest accuracy. An overlapping sliding window scheme also resulted in further performance improvements for $T = \{1, 5, 10\}$ s. It also increases the size of training data.

**Table 7.** Performance of the optical flow stream individually trained with different sliding window configurations.

Configuration	AUC	Accuracy	Precision	Recall	Specificity
*T* = 1 s, $\tau = 1$ s	95.2	88.5	89.3	87.5	89.5
*T* = 5 s, $\tau = 1$ s	**97.4**	**92.2**	**91.7**	**92.8**	**91.6**
*T* = 5 s, $\tau = 5$ s	95.9	89.7	90.6	88.5	90.8
*T* = 10 s, $\tau = 1$ s	96.2	88.2	88.2	91.5	87.8
*T* = 10 s, $\tau = 10$ s	95.5	88.5	87.2	90.4	86.7

*Note*. All performance values are expressed as a percentage.

For the intervention detection task, false positives were found when (1) the infant was very active or crying; (2) the position of the camera was adjusted by clinical staff; (3) lighting conditions changed abruptly when fluorescent lights were switched on or off, or window blinds were opened or closed; (4) daylight illumination changed quickly as the sky went from clear to cloudy, and vice versa; (5) clinical staff were near the incubator causing shadows to be cast on the infant; and (6) the incubator was touched and then shaken when clinical staff came to check the equipment. These scenarios created large movements, abrupt changes in the computation of optical flow, and thus affected the performance of the algorithms. Several false positives that involve intensity changes in the entire field of view can be filtered out by using a post-processing technique such as intensity thresholding.

False negatives usually occurred when (1) parents calmly held the infant in their hands during their visits; (2) clinical staff provided stimulation by touching the infant, so their hands stayed still inside the incubator for a short time; (3) clinical staffs hands were off from the baby during the intervention; or (4) clinical staff quietly held a timer during manual respiratory counting. The errors were likely to have been caused by small changes in optical flow during these scenarios, such that the intervention detection network misinterpreted an intervention period as non-intervention.

Our deep learning framework can perform all the necessary processing steps prior to the estimation of vital signs. For each video frame, the network checks for the presence of a patient in front of the camera and segments skin regions. On the Geforce GTX Titan 6GB, the patient detection and skin segmentation network could process a $512\times384$ image at a rate of 10.9 images per second if a subject was found in the image, and at a rate of 16.4 images per second if the subject was absent from the image. Real-time performance could be achieved for video processing with dual GPUs, a lower sampling rate or a smaller image size. For every second, the intervention detection network identifies the presence of intervention in a video segment. The computation of an optical flow vector in a 5 s window segment takes 0.35 s. The intervention detection network takes 0.03 s to process the input of skin confidence maps and optical flow vectors. Hence, the whole processing time for each 5 s segment is 0.38 s.

In our setup, a video camera was positioned inside the incubator’s chamber through a specifically-drilled hole in the top plastic panel of the incubator’s canopy. The video camera can also be positioned at the side or the corner of the incubator’s chamber. We expect our deep learning framework could produce similar results for different incubator and camera set-ups, provided that an infant was not positioned off-center of the camera’s field of view. Our deep learning models could be improved by additional training data on different camera set-ups.

**Figure 9.**
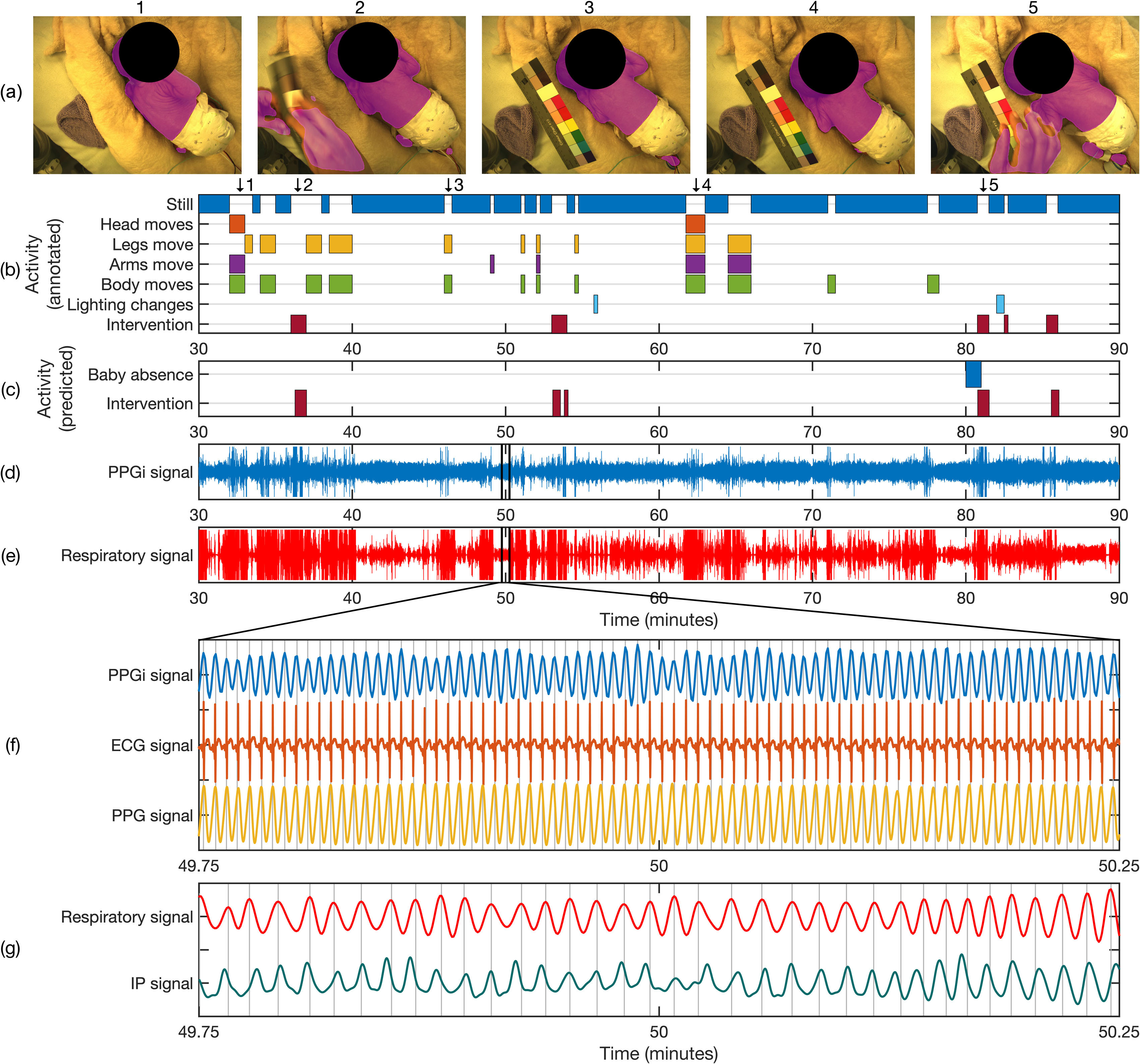
Extraction of PPGi and respiratory signals from segmented skin area. (a) Video frames with segmented skin area provided by our proposed framework. (b) Timeline of patient activities over a 60 min segment for a typical recording session, manually annotated over a minute-by-minute basis. (c) Timeline of predicted time periods for infant absence and clinical intervention provided by the proposed algorithms. (d) 60 min time series of the PPGi signal extracted from the mean pixel intensity of the entire segmented skin region in the green channel. (e) 60 min time series of the respiratory signal extracted from the area of the entire segmented skin region. (f) Comparison of non-contact PPGi, contact ECG, and contact PPG signals for the area highlighted in (d). Each signal contains 78 peaks corresponding to a heart rate of 156 beats min^−1^. (g) Comparison of non-contact respiratory and contact impedance pneumographic (IP) signals for the area highlighted in (e). Each signal contains 35 peaks corresponding to a respiratory rate of 70 beats min^−1^.

**Figure 10.**
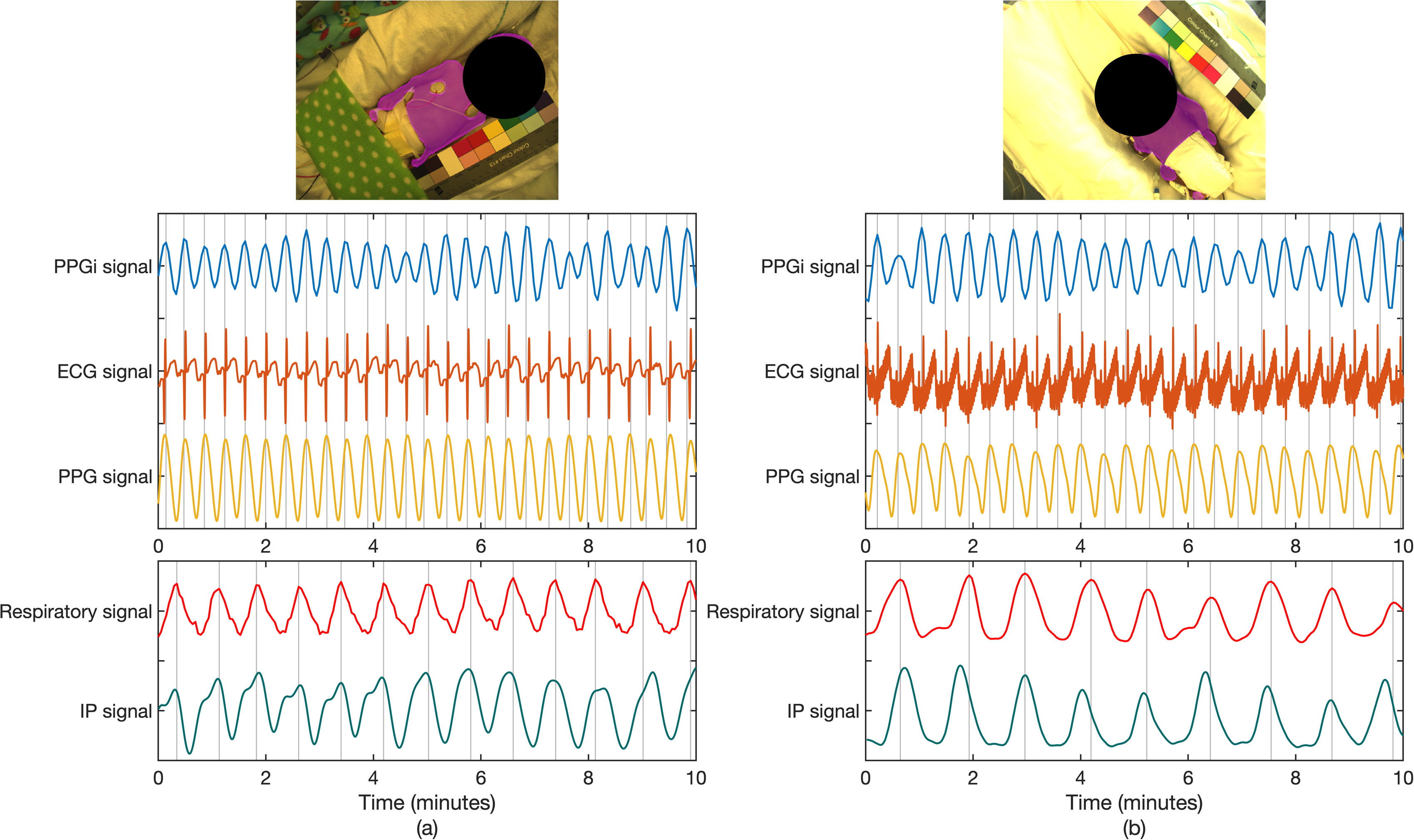
Comparisons of non-contact and contact signals from different subjects. (a) Signals extracted from a mixed-race subject. Each cardiac signal contains 27 peaks corresponding to a heart rate of 162 beats min^−1^. Each respiratory signal contains 13 peaks corresponding to a respiratory rate of 78 breath min^−1^. (b) Signals extracted from a subject with dark skin. Each cardiac signal contains 24 peaks corresponding to a heart rate of 144 beats min^−1^. Each respiratory signal contains nine peaks corresponding to a respiratory rate of 54 breath min^−1^.

Using our deep learning framework, pulsatile PPGi signals could be derived by spatially averaging the intensity of all pixels in the whole skin area for each frame in the video. Respiratory signals could be derived by measuring changes on the properties of the skin area, such as center of mass, area or perimeter. Jorge *et al* (2018) showed that respiratory rate in pre-term infants can be derived from the analysis of changes in the geometric properties of the segmented skin area according to the movement of the upper body during breathing. Figure 9 shows an example of the extraction of non-contact PPGi and respiratory signals from the segmented skin area in comparison to the signals acquired with standard contact techniques. Figure 10 shows comparisons of non-contact and contact signals from different infants. The use of the entire skin area could allow the estimation of heart rate and respiratory rate to be performed automatically in challenging clinical settings, e.g. different skin tone and posture changes.

## 8. Conclusion

It has been previously demonstrated that vital signs (such as heart rate, respiratory rate and peripheral oxygen saturation) can be derived from the measurement of the ambient light reflected from the skin using a video camera. This paper proposed deep learning methods for determining suitable time periods and skin ROIs for continuous non-contact vital sign monitoring in a real hospital environment. The proposed models achieved high performance and demonstrated robustness against pose variations and illumination changes.
